# Descriptive Patterns of Deafness Among Pre-School Saudi Children Aged Two to Five Years Visiting Neurology Clinic From 2012 to 2017

**DOI:** 10.7759/cureus.10893

**Published:** 2020-10-11

**Authors:** Salah Elmalik, Saleh Alshawi, Ahmed Moraya AlQahtani, Hassan S AlShammasi, Ahmed Alruwaili, Ahmad Aldughaim, Saleh Abdullah Alkhalifa

**Affiliations:** 1 Physiology, King Saud Medical City, Riyadh, SAU; 2 Medicine, College of Medicine, King Saud University, Riyadh, SAU

**Keywords:** sensorineural hearing loss, brain audiometry evoked potential, decibel, saudi arabia, pre-school children

## Abstract

Background

Early childhood years are very important and crucial periods for developing different developmental milestones. Hearing loss is considered to be one of the most commonly detectable problems, which often goes unnoticed or not given proper attention due to the lack of screening modalities or the inability of parents or guardians to recognize it in early stages. Therefore, it is necessary to determine the pattern of differences pertaining to hearing loss among pre-school children of various age groups to better approach this issue in a systemic and fundamental manner, so that better care and treatment can be provided to children suffering from deafness.

Methods

This study involved a descriptive, retrospective chart review in two hospital settings, and it was conducted at the department of physiology (neurophysiology) of King Abdulaziz and King Khalid University Hospitals at the King Saud University (KSU) in Riyadh during the period of 2012-2017. A total of 324 pre-school Saudi children from the age of two to five years were involved and tested by brainstem auditory evoked potentials (BAEPs) to assess deafness.

Results

A total of 324 patients underwent the BAEP test; of them, 199 (61.4%) were males and 125 (38.6%) were females. Regarding the age groups, the most common age group was that of two-year-olds with 117 (36.1%) participants, followed by three-year-olds with 80 (24.7%) children, four-year-olds with 73 (22.5%) patients, and five-year-olds with 54 (16.7%) participants. Furthermore, there were 220 (67.9%) patients with sensorineural hearing loss (SNHL), 92 (28.4%) with conductive hearing loss (CHL), four (1.2%) with mixed hearing loss (MHL), and eight (2.5%) with normal audiometry. The normal hearing threshold was determined to be 20 dB, and the mean value for the hearing threshold of the SNHL in the right ear was found to be 43.45 ± 25.85, while the left-ear mean value was 44.54 ± 28.78. The mean value of the hearing threshold in CHL of the right ear was 50.96 ± 22.23, while that of the left ear was 47.85 ± 22.74. Lastly, the mean value of the hearing threshold in MHL of the right ear was 80.00 ± 21.21, while that of the left ear was 73.75 ± 18.87.

Conclusion

SNHL was the most common type (67.9%) of pre-school hearing loss in Saudi Children attending the neurophysiology clinic at KSU hospitals between 2012-2017, while MHL constituted the most severe cases.

## Introduction

Hearing ability is important for children to develop speech and language skills as they grow up. In the past, hearing loss in children often went undetected until the child turned around two years of age, when it would become obvious as they had not started talking yet [1]. Hearing loss is a major public health issue that is often neglected [2]. According to the World Health Organization (WHO), 360 million people worldwide have disabling hearing loss, and 32 million of them are children [3]. Furthermore, 40% of childhood hearing loss is non-preventable, while 60% of childhood hearing loss is due to preventable causes, of which 31% are related to infections, 17% to birth-related causes, 4% to ototoxic medicines, while 8% are associated with other causes [4]. Hearing loss, also known as hearing impairment, is defined as a partial or total inability to hear. Hearing loss may occur in one or both ears. One of the most common ways to measure the auditory profile of pre-school children is the brainstem auditory evoked potential (BAEP) test. It is a non-invasive test to measure the brainstem electrical potentials that occur in response to sound clicks with a minimum recording duration of one hour [5]. The absolute latency of waves I, V, and interpeak latency of I-V are recorded in all subjects. Hearing loss can be classified into three types: sensorineural, conductive, and mixed [6]. The vast majority of people with hearing loss have sensorineural hearing loss (SNHL), and it occurs from a lack of development or damage to either the nerve or the special sensory cells for hearing in the inner ear, known as the hair cells, with variable degrees of severity ranging from mild or moderate to severe or profound [7,8]. Meanwhile, conductive hearing loss (CHL) can be a result of diseases that affect the external ear or middle ear structures. However, it can only be mild or moderate and lacks the severe characteristics of SNHL [9,10]. On the other hand, mixed hearing loss (MHL), which is a combination of both SNHL and CHL, can result from problems affecting either the inner ear, the middle ear, or the outer ear [10].

## Materials and methods

This study involved a cross-sectional chart review in two hospital settings that are managed by one administration and share the same system and patient files. The study was conducted at the department of physiology (neurophysiology) of King Abdulaziz and King Khalid University Hospitals at the King Saud University (KSU) in Riyadh during the period of 2012-2017. A total of 324, out of a potential 522 (95% confidence level and assuming a prevalence rate of 50%), pre-school Saudi children from the age of two to five years were involved and tested by BAEPs to assess deafness. The sample size was collected using a systematic random sampling technique. All pre-school children who underwent BAEPs were included, while those with incomplete files or examinations were excluded. All personal information and the identity of the patients taken from the medical records were kept confidential. Descriptive data distribution and frequencies were measured using Microsoft Excel 2016 (Microsoft Corporation, Redmond, WA) and were divided into tables and graphs. The level of severity was measured according to the range of decibels: light severity was considered to range from 26 dB to 40 dB, moderate from 41 dB to 61 dB, severe from 61dB to 80dB, and profound severity was considered to correspond to a value of ≥81 dB [10].

## Results

A total of 324 patients underwent the BAEP test. Regarding the age of participants, children aged two years formed the largest group, accounting for 117 (36.1%) participants, followed by 80 children aged three years (24.7%), 73 (22.5%) aged four years, and 54 (16.7%) five-year-olds. Furthermore, there were more male children than female children, with 199 (61.4%) male children and 125 (38.6%) female children (Table 1).

**Table 1:** Sociodemographic characteristics (N=324)

Characteristics	Frequency (%)
Age in years	
2	117 (36.1)
3	80 (24.7)
4	73 (22.5)
5	54 (16.7)
Gender	
Male	199 (61.4)
Female	125 (38.6)

**Figure 1:**
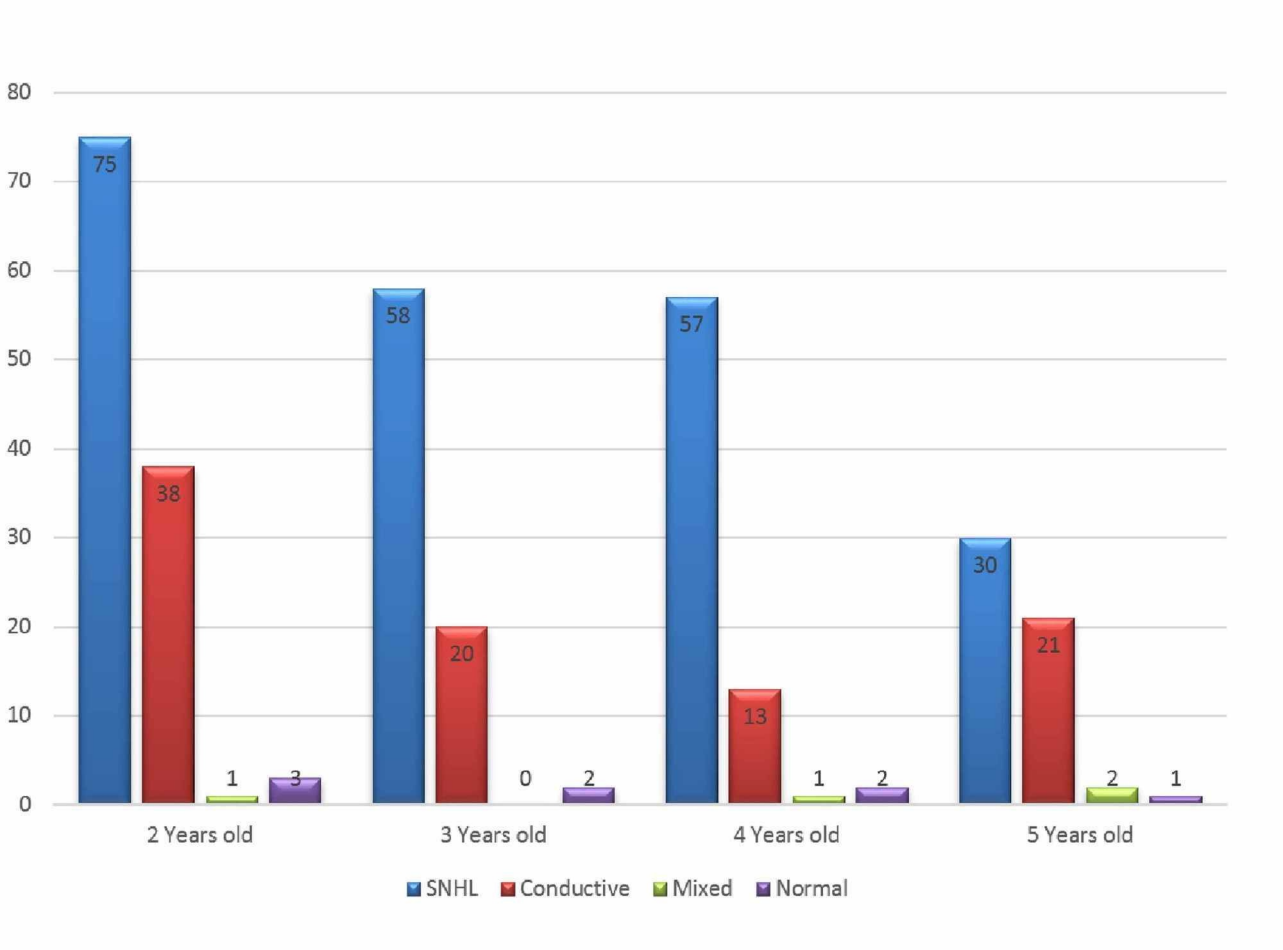
Hearing loss patterns in children aged two to five years SNHL: sensorineural hearing loss

**Figure 2:**
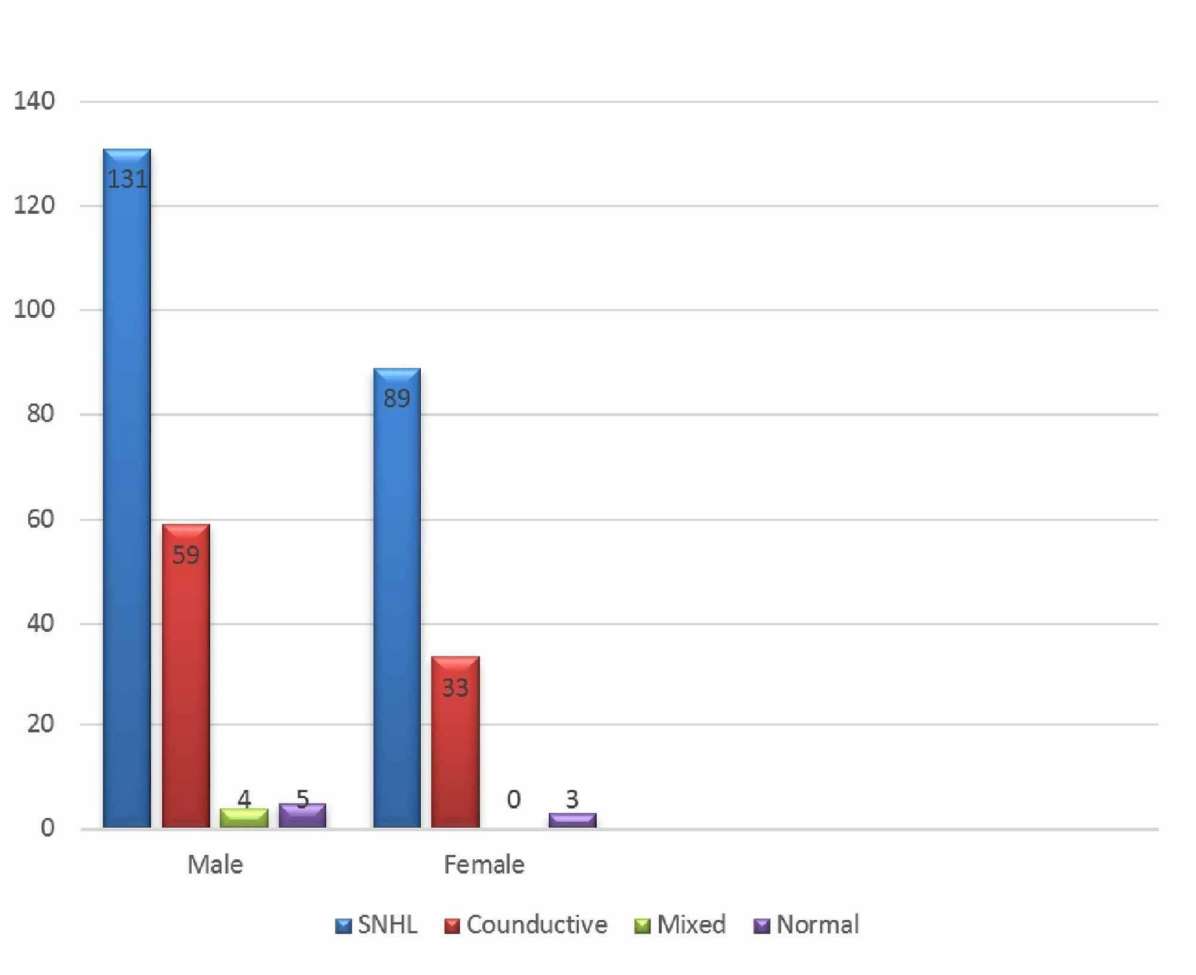
Hearing loss types by gender SNHL: sensorineural hearing loss

Regarding the distribution of the types of hearing loss, the most commonly reported hearing loss was found to be SNHL (220; 67.9%), followed by CHL (92; 28.4%). Meanwhile, MHL was the least commonly reported hearing loss, with only four (1.2%) children affected by it, while eight BAEP tests came back normal (2.5%) (Table 2). Moreover, SNHL showed a steady decrease with increasing age, with 75 reported cases in the two-year-old group, which decreased to almost half that amount, reaching 30 reported cases, in the five-year-old group (Figure 1). In addition, SNHL had a male predominance, with 131 reported cases in males compared to 89 cases reported in females. Interestingly, MHL was reported only in males with four documented cases, while it was absent in females (Figure 2).

**Table 2:** Frequencies of hearing loss types (N=324) SNHL: sensorineural hearing loss; CHL: conductive hearing loss; MHL: mixed hearing loss

Hearing loss type	Frequency (%)
SNHL	220 (67.9)
CHL	92 (28.4)
MHL	4 (1.2)
Normal	8 (2.5)

The severity of the hearing loss was recorded in decibels and was reported as mean values and standard deviations (SD). The severity was found to be moderate in both ears with SNHL and CHL types. The right-ear SNHL mean value was found to be 43.45 ± 25.85, while in the left ear it was fairly similar at 44.54 ± 28.78. Furthermore, the CHL mean value in the right ear was 50.96 ± 22.23 compared to a slightly lower value in the left ear at 47.85 ± 22.74. On the other hand, MHL was considered severe due to a mean value of 80.00 ± 21.21 in the right ear and 73.75 ± 18.87 in the left ear (Table 3). However, no cases of profound or mild severity were found among any of the groups.

**Table 3:** Degree of severity (N=324) SNHL: sensorineural hearing loss; CHL: conductive hearing loss; MHL: mixed hearing loss; SD: standard deviation

Type	Mean (SD)
SNHL right ear moderate	43.45 ± 25.85
SNHL left ear moderate	44.54 ± 28.78
CHL right ear moderate	50.96 ± 22.23
CHL left ear moderate	47.85 ± 22.74
MHL right ear severe	80.00 ± 21.21
MHL left ear severe	73.75 ± 18.87

## Discussion

SNHL is the most commonly reported hearing deficit among children and infants, consisting of around 4,000 reported cases in newborns with severe to profound SNHL [11,12]. Meanwhile, at least three out of 10,000 school children have reported mild to moderate severity of SNHL where it was considered as a late-onset diagnosis due to the fact that it was not severe enough to be noted. However, school performance was affected by it in up to 15% of the pre-school children, which can lead to social implications later on in their lives [12]. BAEP is considered one of the most reliable tests used in groups where normal audiometry is not possible or unachievable. It has been shown to have good reliability in assessing the cochlea and caudal neural brainstem pathway with a reported sensitivity of up to 90% and specificity of up to 100% [13,14]. Therefore, it was our modality of choice in assessing the severity of hearing loss among children.

Brookhouser has stated that in developing countries, two per 1,000 pre-school children exhibit an SNHL of 50 dB, while 0.5 per 1,000 children exhibit a more severe form corresponding to ≥75 dB [15]. In our study, we found that the severity of SNHL (67.9%) was around 45 dB with a decreasing trend with increasing age. Meanwhile, CHL can be further divided into acquired or congenital types. Regardless, studies have shown that when compared to SNHL, it has less effect on school and social performance, and is less common in pre-school groups [12,16]. However, CHL is more often caused by congenital causes where it requires more investigations to pinpoint the underlying causes and treat accordingly, unlike SNHL, which is easily manageable with conventional hearing aids [17]. Unlike our study findings, the study conducted by Al-Rowaily et al. in 2012 among the pre-school population in Riyadh found that CHL was the most common cause of deafness (84.4%), with the majority of cases being found in females. Moreover, they found that the etiology of CHL was attributed to otitis media with effusion (34.9%) as the most common cause, followed by chronic otitis media (23.3%) [18]; when compared to our study, it shows almost the opposite trend, making more screening programs crucial to determine the correct causes and trends of hearing loss among pre-school children. Lastly, MHL has often been linked to many syndromes, diseases, and malformations in the literature [19,20]. Even though our study found very few incidences of MHL with only four cases, those were transferred for further evaluation.

One of the limitations of our study is that we did not further explore the causes of SNHL or CHL. In addition, since our study was only descriptive in nature and did not emphasize on the statistical arm, our sole aim was to shed light on the importance of developing a screening program among the age groups we discussed, by reporting the most common types of hearing losses and their distribution and severity among pre-school children.

## Conclusions

The most common cause of hearing loss found in our study was SNHL. Meanwhile, the severity of the hearing loss was found to be decreasing with increasing age, and the most severely documented decibel level by BAEP was in the MHL group. We recommend that further studies be conducted to explore the etiologies of the conditions, as well as to devise a screening program for children of pre-school age to help in their school and social performance by diagnosing hearing issues and intervening as early as possible.
